# Hepcidin and diabetes are independently related with soluble transferrin receptor levels in chronic dialysis patients

**DOI:** 10.1080/0886022X.2019.1635893

**Published:** 2019-07-11

**Authors:** Luís Belo, Susana Rocha, Maria João Valente, Susana Coimbra, Cristina Catarino, Elsa Bronze-da-Rocha, Petronila Rocha-Pereira, Maria do Sameiro-Faria, José Gerardo Oliveira, José Madureira, João Carlos Fernandes, Vasco Miranda, Alice Santos-Silva

**Affiliations:** a UCIBIO, REQUIMTE, Laboratory of Biochemistry, Department of Biological Sciences, Faculty of Pharmacy, University of Porto, Porto, Portugal;; b CESPU, Institute of Research and Advanced Training in Health Sciences and Technologies (IINFACTS), Gandra-Paredes, Portugal;; c Health Sciences Research Centre, University of Beira Interior, Covilhã, Portugal;; d Hemodialysis Clinic of Felgueiras, CHF, Felgueiras, Portugal;; e Hemodialysis Clinic of Porto, CHP, Porto, Portugal;; f Center for Health Technology and Services Research (CINTESIS), Faculty of Medicine, University of Porto, Porto, Portugal;; g NefroServe Hemodialysis Clinic of Barcelos, Barcelos, Portugal;; h NefroServe Hemodialysis Clinic of Viana do Castelo, Viana do Castelo, Portugal;; i Hemodialysis Clinic of Gondomar, CHD, Gondomar, Portugal

**Keywords:** End-stage renal disease, erythropoiesis, soluble transferrin receptor, hepcidin, iron

## Abstract

**Background:** Soluble transferrin receptor (sTfR) is a biomarker of erythropoiesis, which is often impaired in dialysis patients. The aim of our study was to evaluate sTfR levels in chronically dialyzed patients and assess potential determinants of its levels.

**Methods:** We performed a cross-sectional study by evaluating 246 end-stage renal disease patients undergoing dialysis and 32 healthy controls. Circulating levels of interleukin (IL)-6, C-reactive protein (CRP), tumor necrosis factor (TNF)-α, hepcidin, sTfR, growth differentiation factor 15 (GDF15), and traditional iron metabolism markers were measured, as well as hemogram parameters. Clinical data was obtained from all patients.

**Results:** Compared to controls, patients presented similar values of sTfR, reticulocytes and reticulocyte production index (RPI), and significantly higher levels of IL-6, CRP, ferritin, hepcidin, TNF-α, and GDF15. Iron, transferrin, hemoglobin levels, erythrocyte count, mean cell hemoglobin (MCH), and mean cell hemoglobin concentration (MCHC) values were significantly lower in dialysis group. Within patients, sTfR values were higher in diabetic patients and were positively and significantly correlated with reticulocytes and erythrocytes, RPI, and therapeutic doses of erythropoiesis stimulating agents (ESA) and intravenous iron; and inversely and significantly correlated with circulating iron, ferritin, transferrin saturation, hepcidin, MCH, and MCHC. In multiple linear regression analysis, ESA dose, RPI, serum iron, diabetes, and hepcidin levels were independently associated with sTfR levels in dialysis patients and, thus, with erythropoiesis.

**Conclusion:** Our data suggest that, besides RPI and ESA dose, diabetes and hepcidin are closely related to erythropoiesis in dialysis patients. The influence of diabetes on sTfR levels deserves further investigation.

## Introduction

Transferrin is a plasma glycoprotein that transports iron to cells through the interaction with a specific membrane protein, the transferrin receptor (TfR). The expression and synthesis of TfR is mainly regulated by iron demands, as well as by the interaction of erythropoietin (EPO) with surface EPO receptors on erythroid cells [1].

A truncated soluble form of TfR (sTfR), first described by Kohgo et al. in human serum [2], decreases in case of erythroid hypoplasia and aplastic anemia, increases in erythroid hyperplasia and iron deficiency anemia, and remains relatively unchanged in anemia of chronic diseases [3–5]. Thus, sTfR may reflect erythropoietic activity and iron needs for erythropoiesis. Measurement of sTfR is particularly useful for a differential diagnosis of anemia and for monitoring the erythropoietic response to treatment of the anemia [6]. For instance, in hemodialysis patients under erythropoiesis stimulating agents (ESA) therapy, different studies showed that sTfR provides a promising tool to assess bone marrow erythropoietic activity and iron status [7–9]. Recently, sTfR level has been proposed as a useful marker to diagnose iron deficiency-based anemia in chronic dialysis patients [10]. However, the lack of assay standardization and the wide variety in the cutoff values limits its interpretation in clinical practice [11]. A future application of this parameter also depends on a better understanding on factors modulating its levels.

The number of reticulocytes and the reticulocyte production index (RPI) are also markers of (effective) erythropoiesis. Under an erythropoietic stimulus and adequate concentrations of erythropoietic nutrients, the production of reticulocytes increases to correct anemia, and they are prematurely released from the bone marrow, according to the severity of anemia. Thus, to evaluate RPI, reticulocyte count needs to be corrected for reticulocyte maturation (days in circulation) and for the degree of anemia, provided by hematocrit [12,13]. In case of iron deficiency, post-transcriptional induction of TfR expression in erythroid cells is activated. Thus, when plasma iron is not adequate for erythropoiesis demands, RPI decreases and sTfR rises.

Hepcidin is a major regulator of iron absorption and mobilization from iron storage, for erythropoiesis [14]. It is mainly synthesized in the liver, although, other tissues, such as the kidney, heart, and adipose tissue, can also express this peptide [15]. Hepcidin induces endocytosis and proteolysis of ferroportin on duodenal enterocytes, reducing iron absorption; and on the membrane of macrophages and hepatocytes, reducing the efflux of iron from the major iron stores, for erythropoiesis [16]. Hepatic hepcidin synthesis is up-regulated by inflammation, through interleukin (IL)-6, transferrin saturation, and liver iron levels, while increasing erythropoietin (EPO), erythropoiesis, and hypoxia down-regulate hepcidin synthesis [17]. Some factors secreted by erythroid cells along the erythropoietic process, such as growth differentiation factor 15 (GDF15) and erythroferron, have also been implicated in the suppression of hepcidin expression [17]. Erythropoiesis and inflammation are, therefore, closely linked and are both altered in dialysis patients [10,18]. The contribution for the increase in hepcidin, in these patients, is still poorly clarified and might result from the enhancement in inflammation, from the lack of renal excretion of this peptide or from other unknown factors [19,20]. A negative correlation between hepcidin and sTfR levels was reported in these patients, although, the underlying mechanisms supporting this observation remain to be elucidated [21,22].

It is also known that iron metabolism and glucose homeostasis are tightly interconnected [23] and that diabetes is a major cause of chronic kidney disease (CKD). It has been reported that sTfR levels are positively associated with insulin resistance in men and postmenopausal women [24] and that in chronic hemodialysis patients, hepcidin-25, the bioactive isoform of hepcidin, is positively associated with the presence of diabetes [22]. However, conflicting results exist regarding the association between sTfR and type 2 diabetes [25].

The cross-talk between inflammation, iron metabolism, and clinical data (including diabetes) appears to be particularly complex in dialysis patients. Our aim was to evaluate the potential determinants (patient-, analytical-, and treatment-related) of sTfR levels in chronically dialyzed patients, namely, how diabetes interferes with iron metabolism and erythropoiesis.

## Materials and methods

### Patients

All procedures performed in studies involving human participants were in accordance with the ethical standards of the Ethics Committee of University of Porto and with the 1964 Helsinki declaration, as revised in 2008. Informed consent was obtained from all individual participants included in the study. Two hundred and forty-six end-stage renal disease (ESRD) patients under dialysis therapy for at least 90 days, from five dialysis clinics in the Northern region of Portugal, were included in a cross-sectional study. Patients were clinically evaluated and blood was collected for the analytical studies before the midweek dialysis session. Data regarding demographic characteristics, CKD, medical history, dialysis, and pharmacological prescriptions were collected. Patients with autoimmune disease, active malignancy, and acute or chronic infection were excluded.

Diabetes was defined by the current guidelines [26] or by the use of insulin or oral hypoglycemic agents. Hypertension was defined as a blood pressure ≥ 140/90 mmHg (average pre-dialysis values from the previous month) or by the use of antihypertensive medication.

In order to characterize the analytical changes occurring in dialysis patients, a group of 32 healthy volunteers was selected as control, based on normal hematological and biochemical values, and no history of diseases that could interfere with our analysis.

### ESA and iron therapies

Therapy with recombinant human erythropoietin (rhEPO) and with intravenous iron was based on the European Renal Best Practice Guidelines [27].

Three kinds of ESA were prescribed, including epoetin α (Eprex^®^; IU), epoetin β (Neorecormon^®^; IU), and darbepoetin α (Aranesp^®^; μg). The doses of epoetin (α and β) were converted to standardized equivalent doses of darbepoetin α, according to the World Health Organization (WHO) daily-defined dose (DDD); in accordance, 1000 IU of epoetin are equivalent to 4.5 μg of darbepoetin α (conversion factor: 222:1; http://www.whocc.no/atc_ddd_index/). Patients on iron therapy used iron sucrose (Venofer^®^).

#### Assays

Blood samples, collected immediately before the dialytic procedure, were processed within 2 h. Blood was collected to tubes with and without anticoagulant (K_3_-EDTA), to obtain whole blood, plasma, and serum. Aliquots of plasma and serum were immediately stored at −80 °C until assayed.

Leukocyte, platelet and erythrocyte counts, hematocrit, hemoglobin concentration, and hematimetric indices [mean cell volume (MCV), mean cell hemoglobin (MCH), and mean cell hemoglobin concentration (MCHC)] were measured by using an automatic blood cell counter (Sysmex K1000; Sysmex, Hamburg, Germany). Reticulocytes were quantified by microscopic counting on blood smears, after vital staining with New methylene blue (Reticulocyte stain; Sigma-Aldrich Co. LLC. St. Louis, MO, USA). The reticulocyte production index (RPI), an appropriate way to measure the effective red blood cell (RBC) production, was calculated by the formula: [(reticulocyte %/maturation time of RBC)*(hematocrit/0.45)], where the maturation time of RBC (days of circulating blood reticulocytes released from the bone marrow) was 1 for hematocrit values between 36% and 45%, 1.5 for hematocrit values between 26% and 35%, 2 for values between 16% and 25%, and 2.5 for values lower than 15% [13].

Serum iron concentration was determined using a colorimetric method (Iron, Randox Laboratories Ltd., North Ireland, UK), whereas serum ferritin and serum transferrin were measured by immunoturbidimetry (Ferritin, Randox Laboratories Ltd., North Ireland, UK; Transferrin, Randox Laboratories Ltd., North Ireland, UK). Transferrin saturation (TS) was calculated by the formula: TS (%) = 70.9 × serum iron concentration (µg/dL)/serum transferrin concentration (mg/dL).

Plasma levels of IL-6, hepcidin, sTfR, tumor necrosis factor (TNF)-α and GDF15, were evaluated by using standard commercially available enzyme linked immunosorbent assays [Human IL-6 Quantikine HS ELISA Kit, Human Hepcidin Quantikine ELISA Kit, Human Soluble Transferrin Receptor Quantikine IVD ELISA Kit and Human TNF-α Quantikine HS ELISA Kit, R&D Systems, Minneapolis, MN, USA, Human GDF-15 ELISA Kit (ab155432), Abcam, Cambridge, UK, respectively]. C-reactive protein (CRP) was evaluated by immunoturbidimetry, using commercially available kit [CRP (Latex) High-Sensitivity, Roche Diagnostics, Basel, Switzerland].

### Statistical analysis

Kolmogorov–Smirnov analysis was used to test if the results were normally distributed. Those variables showing normal distribution are presented as mean ± standard deviation (SD) and those non-normally distributed are presented as median (interquartile range). Differences between groups were tested using chi-squared test and Fisher’s exact test for categorical variables; for continuous variables, comparisons between two groups were performed using Student’s unpaired *t*-test or Mann–Whitney *U* test; for assessing circadian variations (comparison of more than two groups), we used one-way ANOVA supplemented with Bonferroni *post hoc* test (with variables respecting a Gaussian distribution). Adjustment for confounding factors (e.g., BMI or age) was performed using analysis of covariance (variables respected a Gaussian distribution).

The strength of the association between the variables was estimated by Pearson correlation coefficient, after log transformation of the variables (whenever necessary). To evaluate the contribution of different variables to sTfR levels, multiple regression analysis was performed, using stepwise selection, with an entry criteria of *p* < .05.

Statistical analysis was performed using the IBM Statistical Package for Social Sciences (SPSS, version 24.0, Chicago, IL, USA) for Windows. Statistical significance was accepted at *p* less than .05.

## Results

### Demographic and clinical data of patients and controls

We studied 246 ESRD patients under dialysis therapy and 32 controls with similar gender distribution and body mass index (BMI) values. Patients presented higher age and systolic blood pressure, and lower diastolic blood pressure values, compared to controls (Table 1).

**Table 1.** Demographic and clinical data in controls and dialysis patients.

	Controls (*n* = 32)	Patients (*n* = 246)	*P*
Gender, *n* (%)			
Male	13 (40.6)	134 (54.5)	.187
Female	19 (59.4)	112 (45.5)	
Age (years)	56.3 (53.8–59.1)	71.0 (59.7–79.5)	<.001
BMI (Kg/m^2^)	25.0 ± 3.1	25.6 ± 4.7	.341
Blood pressure (mmHg)			
Systolic	122.9 ± 10.1	137.9 ± 21.5	<.001
Diastolic	80.6 ± 7.8	62.9 ± 12.9	<.001
Most prevalent comorbidities, *n* (%)			
Diabetes		98 (39.8)	
Hypertension		153 (62.2)	
Cause of renal failure, *n* (%)			
Diabetes	–	87 (35.4)	–
Hypertension		34 (13.8)	
Polycystic kidney disease		17 (6.9)	
Chronic glomerulonephritis		18 (7.3)	
Other		39 (15.9)	
Undetermined		51 (20.7)	
Dialysis vintage (years)	–	3.87 (1.79–7.48)	–
Dialysis therapy, *n* (%)			
High-flux hemodialysis	–	30 (12.2)	–
Online hemodiafiltration		216 (87.8)	
Vascular access, *n* (%)			
Arteriovenous fistula	–	199 (80.9)	–
Arteriovenous graft		12 (4.9)	
Central venous catheter		35 (14.2)	
Dialysis efficacy			
URR (%)	–	79.0 (75.8–83.0)	–
Kt/V		1.81 ± 0.32	
eKt/V		1.62 ± 0.28	
Ultrafiltration volume (L)		2.3 (1.7–2.9)	
Prescription of ESA			
*n* (%)	–	210 (85.4)	–
Darbepoetin α / Epoetin α or β		156 / 54	
ESA dose (µg/Kg/week)		0.37 (0.20–0.63)	
Iron therapy			
*n* (%)	–	161 (65.4)	–
mg/week		50.0 (25.0–60.0)	

Values are presented as mean ± SD or median (interquartile range), unless otherwise indicated.

BMI: body mass index; URR: urea reduction ratio; ESA: erythropoiesis stimulating agents. ESA dose was calculated by converting the doses of epoetin (α and β) to standardized equivalent doses of darbepoetin α: according to the World Health Organization (WHO) daily-defined dose (DDD).

Patients were under therapeutic dialysis three times per week, for 3–5 h, and were under treatment for a median period of 3.87 years. The dialysis sessions were performed during three different periods of the day, starting in the morning (7:30 am; *n* = 82), nearly midday (00:30 pm; *n* = 88), and in the evening (5:30 pm; *n* = 76).

Patients were under online hemodiafiltration (HDF; 87.8%) or under high-flux hemodialysis (12.2%) treatment (Table 1), and used high-flux polysulfone FX-class dialyzers (1.4–2.2 m^2^) of Fresenius (Bad Homburg, Germany). The most prevalent vascular access used by patients was arteriovenous fistula (80.9%), followed by central venous catheter (14.2%) and arteriovenous graft (4.9%). Causes of renal failure in the studied patients were diabetes mellitus (*n* = 87), arterial hypertension (*n* = 34), glomerulonephritis (*n* = 18), polycystic renal disease (*n* = 17), other diseases (*n* = 39), and uncertain etiology (*n* = 51).

A total of 153 patients (62.2%) had arterial hypertension and 98 (39.8%) were diabetic; 77 patients, out of the 153 hypertensive and of the 98 diabetic, were both diabetic and hypertensive. Some patients were under pharmacological treatment with ESA (85.4%), intravenous iron (65.4%), antihypertensives (38.2%), antidiabetics (37.4%), statins (51.6%), antiplatelets (46.7%), and with oral anticoagulants (14.6%).

Diabetic patients (*n* = 98) were treated with insulin (*n* = 70; 71.4%), oral hypoglycemic agents (*n* = 9; 9.2%), or with both insulin and oral agents (*n* = 13; 13.3%); 6 diabetic patients (6.1%) were not medicated with antidiabetics.

Diabetic (*n* = 98) and nondiabetic (*n* = 148) patients presented similar (*p* > .05) gender distribution (48.0 vs. 43.8% females), age [71 (62–77) vs. 71 (57–81) years], diastolic blood pressure [63 ± 12 vs. 63 ± 14 mmHg], dialysis modality (85.7 vs. 89.2% patients under online HDF), and therapeutic dose of ESA [0.38 (0.20–0.63) vs 0.35 (0.21–0.65) µg/Kg/week, *n* = 85 vs. *n* = 125] and iron [50 (25–95) vs. 45 (25–50) mg/week, *n* = 69 vs. *n* = 92]. Diabetic patients presented higher BMI [27.4 ± 4.1 vs. 24.4 ± 4.7 Kg/m^2^, *p* < .001] and systolic blood pressure [146 ± 20 vs. 133 ± 21 mmHg, *p* < .001], compared with nondiabetic patients.

### Hematological and biochemical data of patients and controls

Compared to controls, dialysis patients presented similar values for reticulocytes, RPI, sTfR, and MCH and significantly higher values for leukocytes and circulating levels of IL-6, CRP, TNF-α, GDF15, ferritin, and hepcidin. Hemoglobin concentration, hematocrit, erythrocyte, and platelet counts, MCHC values, serum iron, transferrin, and transferrin saturation were significantly lower in patients (Table 2). Results remained statistically significant after adjustment for age as a confounding factor.

**Table 2.** Hematological and biochemical data in controls and dialysis patients.

	Controls (*n* = 32)	Patients (*n* = 246)	*P*
Hematological Data			
Erythrocytes (×10^12^/L)	4.59 (4.29–5.04)	3.73 (3.48–4.00)	<.001
Hemoglobin (g/dL)	13.8 (13.1–15.6)	11.4 (10.7–12.1)	<.001
Hematocrit (%)	41.2 (39.4–45.8)	35.1 (33.1–37.2)	<.001
Reticulocytes (×10^9^/L)	39.8 (30.6–47.8)	40.1 (26.8–58.0)	.793
RPI	0.79 (0.61–0.96)	0.69 (0.48–1.06)	.190
MCV (fL)	90.9 ± 3.3	94.5 ± 5.4	<.001
MCH (pg)	30.7 (29.5–31.7)	30.8 (29.8–31.9)	.676
MCHC (g/dL)	33.6 (33.2–34.0)	32.5 (31.8–33.1)	<.001
Platelets (×10^9^/L)	273 (213–331)	195 (158–231)	<.001
Leukocytes (×10^9^/L)	5.3 (4.6–6.5)	6.2 (5.2–7.5)	.011
Iron metabolism markers			
Iron (µg/dL)	105.5 (86.0–131.2)	55.0 (45.0–74.0)	<.001
Transferrin (mg/dL)	261.6 (236.4–288.4)	187.0 (164.8–216.8)	<.001
Transferrin saturation (%)	29.4 (22.4–37.6)	21.6 (15.9–27.7)	<.001
sTfR (nM)	23.6 (16.5–28.0)	22.0 (16.8–28.1)	.775
Ferritin (ng/mL)	88.0 (44.2–157.2)	303.5 (176.0–456.5)	<.001
Hepcidin (ng/mL)	20.4 (11.7–35.6)	76.5 (40.4–137.7)	<.001
Inflammatory markers			
IL-6 (pg/mL)	1.12 (0.74–1.62)	4.09 (2.64–7.47)	<.001
hs-CRP (mg/dL)	0.15 (0.04–0.26)	0.36 (0.18–0.77)	<.001
TNF-α (pg/mL)	0.81 (0.68–1.07)	3.31 (2.65–4.45)	<.001
GDF15 (pg/mL)	970 (810–1090)	11,450 (8600–14,290)	<.001

Values are presented as mean ± SD or median (interquartile range).

RPI: reticulocyte production index; MCV: mean cell volume; MCH: mean cell hemoglobin; MCHC: mean cell hemoglobin concentration; sTfR: soluble transferrin receptor; IL-6: interleukin-6; hs-CRP: high sensitivity C-reactive protein; TNF: tumor necrosis factor; GDF15: growth differentiation factor 15.

No significant differences were observed for hematological or biochemical variables between samples collected from patients at different periods of the day (morning, midday, and evening), except for transferrin saturation that was higher in patients treated in the morning (Supporting Information Table 1).

**Figure 1.**
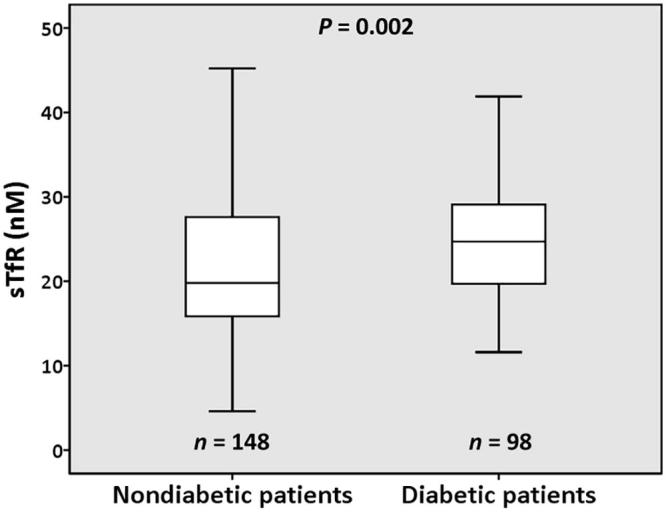
Plasma levels of soluble transferrin receptor (sTfR) in diabetic and nondiabetic chronic dialysis patients. The boxes represent the interquartile range (IQR), with the upper and lower edges of the boxes representing the 75th and 25th percentiles, respectively. The central horizontal lines within the boxes represent median levels for each group. The vertical whiskers above and below the boxes represent the range of outlying data points up to 1.5 times the IQR; they extend from the box to the highest and lowest values, excluding outliers.

Diabetic patients presented higher erythrocyte, reticulocyte and leukocyte counts, higher RPI values (Table 3) and sTfR levels (Figure 1) compared with nondiabetic patients; MCV, MCH, and CRP were lower for diabetic patients (Table 3).

**Table 3.** Hematological and biochemical data in diabetic and nondiabetic dialysis patients.

	Non diabetic patients (*n* = 148)	Diabetic patients (*n* = 98)	*P*
Hematological data			
Erythrocytes (× 10^12^/L)	3.70 (3.40 –3.96)	3.84 (3.54–4.09)	.007
Hemoglobin (g/dL)	11.4 (10.6–12.1)	11.5 (10.9–12.2)	.301
Hematocrit (%)	34.8 (32.5–37.2)	35.5 (33.9–37.2)	.103
Reticulocytes (×10^9^/L)	38.5 (26.1–54.2)	45.9 (27.2–67.8)	.042*
RPI	0.61 (0.46–0.90)	0.78 (0.50–1.23)	.010
MCV (fL)	95.3 ± 5.4	93.3 ± 5.4	.004
MCH (pg)	31.2 (30.1–32.1)	30.5 (29.3–31.4)	.002
MCHC (g/dL)	32.5 (31.9–33.2)	32.4 (31.7–33.1)	.770
Platelets (×10^9^/L)	194 (152–232)	200 (165–232)	.307
Leukocytes (×10^9^/L)	5.8 (5.0–7.2)	6.7 (5.6–8.0)	<.001
Iron metabolism markers			
Iron (µg/dL)	54.0 (45.0–75.0)	60.0 (46.0–73.0)	.631
Transferrin (mg/dL)	184.0 (163.2–213.0)	196.0 (165.8–224.0)	.163
Transferrin saturation (%)	21.2 (15.6–28.0)	22.7 (16.2–27.0)	.963
sTfR (nM)	19.8 (15.8–27.6)	24.7 (19.6–29.4)	.002
Ferritin (ng/mL)	328.0 (177.2–469.8)	285.0 (173.8–448.5)	.247
Hepcidin (ng/mL)	78.6 (43.2–139.0)	73.0 (34.1–126.4)	.246
Inflammatory markers			
IL-6 (pg/mL)	4.07 (2.62–7.82)	4.10 (2.63–6.88)	.802
hs-CRP (mg/dL)	0.42 (0.19–0.87)	0.28 (0.12–0.65)	.023
TNF-α (pg/mL)	3.40 (2.70–4.59)	3.27 (2.44–3.94)	.089
GDF15 (pg/mL)	11,300 (8400–14,170)	12,120 (9040–14,610)	.186

Values are presented as mean ± SD or median (interquartile range).

RPI: reticulocyte production index; MCV: mean cell volume; MCH: mean cell hemoglobin; MCHC: mean cell hemoglobin concentration; sTfR: soluble transferrin receptor; IL-6: interleukin-6; hs-CRP: high sensitivity C-reactive protein; TNF: tumor necrosis factor; GDF15: growth differentiation factor 15.

*Significance was lost (*p* = .083) after adjustment for BMI.

Among diabetic patients, no statistically significant differences were found for the studied analytical variables between those treated or not treated with insulin, apart from transferrin that was lower in insulin treated group (Supporting Information Table 2).

**Figure 2.**
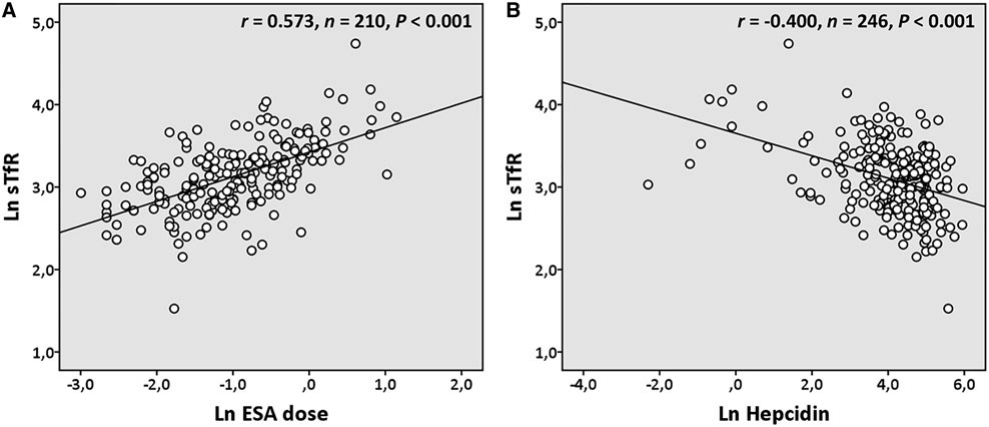
Correlations between plasma levels of soluble transferrin receptor (sTfR) with dose of erythropoiesis stimulating agents (ESA; panel A) and plasma levels of hepcidin (panel B) in chronic dialysis patients. Results were log transformed prior to analysis. *r*: Pearson correlation coefficient.

The values of sTfR values in dialysis patients correlated positively and significantly with reticulocytes (*r* = 0.447, *n* = 246, *p* < .001), erythrocytes (*r* = 0.205, *n* = 246, *p* = .001), RPI (*r* = 0.445, *n* = 246, *p* < .001), hematocrit (*r* = 0.137, *n* = 246, *p* = .031), dose of ESA (Figure 2(A)), and of intravenous iron (*r* = 0.196, *n* = 161, *p* = .013); and were inversely and significantly correlated with hepcidin (Figure 2(B)), iron (*r* = −0.447, *n* = 246, *p* < .001), ferritin (*r* = ‒0.228, *n* = 246, *p* < .001), transferrin saturation (*r* = ‒0.281, *n* = 246, *p* < .001), MCH (*r* = ‒0.381, *n* = 246, *p* < .001), and MCHC (*r* = ‒0.451, *n* = 246, *p* < .001). In multiple linear regression analysis, ESA dose, the presence of diabetes, RPI, circulating iron, and hepcidin remained statistically associated with sTfR values (Table 4).

**Table 4.** Main determinants of soluble transferrin receptor (sTfR) levels in chronic dialysis patients by multiple linear regression analysis.

Dependent variable	Model	Unstandardized coefficients		Standardized coefficients	*t*	*P*
		B	Std. error	Beta		
Ln sTfR	(Constant)	4.617	0.203		22.698	<.001
	Ln ESA dose	0.205	0.026	0.395	7.746	<.001
	Ln RPI	0.202	0.032	0.297	6.238	<.001
	Ln Hepcidin	‒0.079	0.017	−0.226	‒4.689	<.001
	Ln Iron	‒0.234	0.051	−0.227	‒4.548	<.001
	Diabetes (No/Yes)	0.116	0.040	0.135	2.871	.005

*R*^2^ for multivariable regression model = 0.564.

ESA: erythropoiesis stimulating agents; RPI: reticulocyte production index. Diabetes was entered as a dichotomous variable (0 = nondiabetic; 1 = diabetic). The iron dose was removed from the model, as, in a first analysis, it was far from being included in the model and also limited number of cases included in the analysis. Regression analyses were performed with natural logarithm of skewed variables, including the dependent variable (sTfR).

We also found that, in dialysis patients, hepcidin levels were significantly and positively correlated with serum iron (*r* = 0.201, *n* = 246, *p* = .002), ferritin (*r* = 0.643, *n* = 246, *p* < .001), IL-6 (*r* = 0.147, *n* = 246, *p* = .021), and CRP (*r* = 0.137, *n* = 246, *p* = .032); and negatively correlated with erythrocytes (*r* = ‒0.171, *n* = 246, *p* = .007), reticulocytes (*r* = ‒0.196, *n* = 246, *p* = .002), RPI (*r* = ‒0.171, *n* = 246, *p* = .007), and dose of ESA (*r* = ‒0.239, *n* = 210, *p* < .001).

Also in dialysis patients, IL-6 values correlated with iron metabolism markers: iron (*r* = ‒0.214, *n* = 246, *p* = .001), ferritin (*r* = 0.154, *n* = 246, *p* = .015), and transferrin saturation (*r* = ‒0.159, *n* = 246, *p* = .013); similar correlations were observed between CRP and iron (*r* = ‒0.289, *n* = 246, *p* < .001) and between CRP and transferrin saturation (*r* = ‒0.208, *n* = 246, *p* = .003).

## Discussion

The evaluation of sTfR in study of anemia is a valuable tool, but in ESRD patients under dialysis therapy, who present impaired erythropoiesis, disturbed iron metabolism and enhanced inflammation, and are treated with ESA and/or with iron to correct anemia, its clinical application requires a better understanding on the factors modulating its levels. In the present study, we found that ESA dose, diabetes, RPI, iron and hepcidin levels are independent determinants of sTfR in chronically dialyzed patients.

It is known that sTfR levels increase with erythropoietic activity and in case of iron deficient erythropoiesis [8,28]. Compared to controls, our patients presented significantly lower hemoglobin concentration and increased values of inflammatory markers (more than twofold); the significant reduction in serum iron, transferrin, and transferrin saturation, alongside with a significant increase in ferritin, suggest a disturbance in iron absorption and mobilization from iron stores, resulting from the increased inflammatory milieu; however, we found similar sTfR values, circulating reticulocytes and RPI (Table 2), suggesting that sTfR reflects erythropoiesis mainly resulting from ESA therapy. In line with this, we found a strong positive correlation of sTfR with ESA dose.

The positive correlation between sTfR and ESA dose in hemodialysis patients has been reported by others [9] and is in line with previous data from our group, describing higher levels of sTfR in nonresponder patients to rhEPO therapy, who received higher doses of EPO, compared to responders [29]. Nonresponder patients also present a functional iron deficiency associated to enhanced inflammation (as compared to responders) that may contribute to blunt erythropoiesis [29]. Thus, despite increased sTfR values, erythropoiesis is still inadequate in these patients.

Patients on dialysis, with (absolute or functional) iron deficiency, need to be medicated with iron for a more adequate erythropoiesis. In spite of the disturbances in iron metabolism, we found that the treatment of patients with *iv* iron seems to assure adequate iron availability for erythropoiesis, to achieve target hemoglobin values, between 10 and 12 g/dL in these patients. Actually, patients presented well hemoglobinized erythrocytes (although, lower than control values), as showed by the MCH and MCHC values. In accordance, we found a significant positive correlation of sTfR values with the dose of intravenous iron.

Several markers of iron metabolism, serum iron, ferritin, transferrin saturation, and hepcidin, were inversely correlated with sTfR levels; of these, only circulating hepcidin and iron were independent determinants of sTfR levels (Table 4). The importance of hepcidin (with a crucial role both in inflammation and iron metabolism) suggests a central involvement of this peptide in regulating iron availability and, thus, erythropoiesis in these patients. Previous studies in dialysis patients also reported a negative correlation between hepcidin and sTfR levels [21,22], but the mechanisms underlying this association are uncertain. On one side, lower hepcidin levels may facilitate iron mobilization and erythropoiesis, increasing sTfR levels. On the other hand, increased erythropoietic activity (higher sTfR levels) may trigger decreased hepcidin-25 synthesis. EPO itself may directly inhibit liver hepcidin synthesis.

EPO and other factors secreted by erythroid cells, such as GDF15, have been proposed to inhibit liver hepcidin expression [17,30]. We found that GDF15 was increased in hemodialysis patients (Table 2), and, thus, may contribute to a repressing effect on hepcidin expression, and to explain the inverse association between ESA dose and hepcidin levels that we found.

In CKD patients, hepcidin levels are increased, mainly due to its retention due to renal failure [19,20]. Although, IL-6 is a major stimulus for hepatic hepcidin synthesis, and inflammation is enhanced in dialysis patients, controversy exists regarding the contribution of inflammation to hepcidin levels in these patients [22,29,31]. Data from the present study showed small (but significant) correlations of the inflammatory markers, IL-6 and CRP, with hepcidin levels, suggesting a slight contribution of inflammation in its regulation in dialysis patients. Serum ferritin presented the highest correlation with hepcidin. High iron stores promote synthesis of hepcidin, to lower iron absorption from the gut. In agreement with this, a previous study from our group performed in a rat model of chronic renal failure, demonstrated that liver iron is a major regulator of hepcidin gene expression [32]. In spite of the repressing effect of ESAs and erythropoiesis products on hepcidin, its level is still very high in ESRD patients on dialysis (twofold the control values), reducing iron absorption and mobilization from iron stores; we can hypothesize that when ferritin reaches a certain threshold, increasing even more hepcidin synthesis, it will allow worsening of anemia, of iron disturbances, and hyporesponse to ESA therapy. Given the importance of hepcidin, recent research has focused particular attention on its modulation. The use of drugs to inhibit hepcidin synthesis is a new promising therapy to treat anemia of CKD [33,34]. A recent study has demonstrated that a higher hepcidin clearance during dialysis was associated with reduced EPO requirement [19].

The association that we found between diabetes and higher sTfR levels is interesting (Figure 1) and is associated with increased number of reticulocytes and RPI in diabetic patients (Table 3). Despite conflicting results in literature about the relation between sTfR levels and type 2 diabetes [25], a recent study performed in adults at high cardiovascular risk demonstrated that elevated sTfR levels are associated with an increased risk for development of type 2 diabetes, in obese subjects [35]. Obesity triggers a chronic low-grade inflammatory state and is often associated with hypoferremia of inflammation [36,37]. Besides other mechanisms, obesity-associated inflammation increases hepcidin synthesis [15,38] and may contribute to functional iron deficiency. In the present study, diabetic and nondiabetic patients showed similar age, iron metabolism markers, and medication with ESA and iron. In contrast with results from another group [22], we did not find increased levels of hepcidin in dialysis diabetic patients, as compared with nondiabetic patients (Table 3), despite higher BMI values.

It has been reported that type 2 diabetic patients treated with insulin present increased levels of sTfR [39,40]. It was also reported that injection of insulin increases sTfR in rats [41]. Insulin is known to promote erythropoiesis [42,43] and iron uptake by fat cells, redistributing TfR from an internal membrane compartment to the cell surface [44]. Thus, a possible explanation for the results that we observed in diabetic patients is the influence of insulin on sTfR levels. Actually, most diabetic patients were treated with insulin (84.7%), and this drug, by inducing an increase in TfR expression, may allow a more adequate use of circulating iron, for erythropoiesis. We found a trend to higher sTfR levels in diabetic patients treated with insulin, compared to patients treated with oral antidiabetics, but these groups were highly asymmetric (*n* = 15 vs. *n* = 93; Supporting Information Table 2). A trend to lower TNF-α values (Table 3) may also contribute to higher sTfR and RPI in diabetic patients. In fact, proinflammatory cytokines including TNF-α are able to inhibit RBC production, namely by preventing EPO-mediated erythropoiesis at early stages [45].

Altogether, our data suggest that raised hepcidin levels in dialysis patients, due to several mechanisms including inflammation(IL-6)-induced synthesis and kidney retention, may contribute to a functional iron deficiency, as showed by the low serum iron and transferrin concentrations. To improve anemia, due to the incapacity of the failing kidneys to produce EPO and to the low circulating iron available for hemoglobin synthesis in the erythropoietic process, dialysis patients need to be treated with ESA and iron. We found that the doses of both, ESA and iron, are significantly and positively correlated with sTfR, which is likely to reflect the increase in erythropoiesis and iron availability for erythropoiesis. It is known that inflammation, the regular treatment with ESA and iron explain the very high values of ferritin in these patients; the increased ferritin values in the liver might contribute to stimulate the synthesis of hepcidin and thereby to decrease the iron available for erythropoiesis. The administration of iron aims to improve anemia, but it also appears to contribute to increase ferritin, as well as hepcidin levels, that may lead to worsening of functional iron deficiency, and, eventually, to hyporesponse to ESA therapy.

The present study presented some limitations. The cross-sectional design allows to access relationships between sets of data but not causal relationships. Due to CKD, which contraindicates several antidiabetic agents, and also due to the advanced stage of diabetes of most patients, a low number of diabetic patients were only treated with oral antidiabetic agents, not enabling proper statistical comparison of sTfR levels between these patients and those under insulin therapy. The same applies to the modality of dialysis, as some (few) patients were under high-flux hemodialysis. Finally, blood samples were collected at different time periods during the day, and daily variations could interfere in the analysis of some variables (e.g., circadian rhythm may interfere with iron-related markers) [46,47]. Even though we observed no major modifications between time of sampling (apart from transferrin saturation), we tried to compensate for this by adjusting the regression models for period of the time of sampling (morning, midday, and evening) and type of dialysis. This did not change our results, and thus we conclude that these factors were not major confounders in our study.

In conclusion, in chronically dialyzed patients, sTfR levels are independently associated with ESA dose, RPI values, the presence of diabetes, iron and hepcidin levels. The inverse association of hepcidin with sTfR suggests that modulation of hepcidin levels may be beneficial for erythropoiesis in these patients. The influence of diabetes on sTfR levels deserves further investigation.

## Supplementary Material

Supplementary Table 2

Supplementary Table 1
