# Supplementary material for: Hepcidin and diabetes are independently related with soluble transferrin receptor levels in chronic dialysis patients
Source: Ren Fail. 2019 Jul 11;41(1):662–72. doi: 10.1080/0886022X.2019.1635893 (PMC6691825; doi:10.1080/0886022X.2019.1635893)
Supplement: Supplementary Table 2 [file IRNF_A_1635893_SM8843.docx]

**Supplementary Table 2** Hematological and biochemical data in diabetic dialysis patients according to insulin therapy

|  | No Insulin (*n* = 15) | Insulin (*n* = 83) | *P* |
| --- | --- | --- | --- |
| Hematological Data |  |  |  |
| Erythrocytes (x 10^12^/L) | 3.80 (3.71 – 4.06) | 3.84 (3.52 – 4.10) | 0.867 |
| Hemoglobin (g/dL) | 11.4 (10.7 – 11.9) | 11.5 (11.0 – 12.3) | 0.418 |
| Hematocrit (%) | 35.2 (33.1 – 37.1) | 35.6 (34.1 – 37.3) | 0.741 |
| Reticulocytes (x 10^9^/L) | 53.3 (32.5 – 80.1) | 42.9 (27.0 – 63.5) | 0.261 |
| RPI | 0.86 (0.68 – 1.73) | 0.78 (0.49 – 1.19) | 0.216 |
| MCV (fL) | 92.6 ± 4.6 | 93.4 ± 5.5 | 0.584 |
| MCH (pg) | 30.4 (28.0 – 30.8) | 30.5 (29.5 – 31.5) | 0.189 |
| MCHC (g/dL) | 32.3 (31.8 – 32.8) | 32.6 (31.7 – 33.3) | 0.171 |
| Platelets (x 10^9^/L) | 186 (162 – 198) | 206 (166 – 235) | 0.131 |
| Leukocytes (x 10^9^/L) | 6.5 (5.6 – 8.3) | 6.8 (5.6 – 7.9) | 0.921 |
| Iron metabolism markers |  |  |  |
| Iron (µg/dL) | 63.0 (45.0 – 84.0) | 60.0 (46.0 – 70.0) | 0.711 |
| Transferrin (mg/dL) | 222.0 (180.0 – 273.0) | 193.0 (164.0 – 223.0) | 0.031 |
| Transferrin saturation (%) | 16.6 (13.2 – 24.8) | 22.8 (18.4 – 28.6) | 0.158 |
| sTfR (nmol/L) | 19.9 (14.2 – 32.5) | 25.3 (20.1 – 28.2) | 0.119 |
| Ferritin (ng/mL) | 311.0 (162.0 – 494.0) | 265.0 (178.0 – 436.0) | 0.836 |
| Hepcidin (ng/mL) | 99.8 (49.1 – 168.4) | 68.4 (33.9 – 120.5) | 0.148 |
| Inflammatory markers |  |  |  |
| IL-6 (pg/mL) | 3.61 (2.33 – 8.56) | 4.49 (2.65 – 6.88) | 0.456 |
| hs-CRP (mg/dL) | 0.25 (0.13 – 3.26) | 0.30 (0.12 – 0.64) | 0.988 |
| TNF-α (pg/mL) | 3.57 (2.94 – 5.23) | 3.13 (2.42 – 3.85) | 0.081 |
| GDF15 (pg/mL) | 12,160 (10,640 – 15,910) | 12,070 (8,840 – 14,530) | 0.505 |

Values are presented as mean ± SD or median (interquartile range). RPI, reticulocyte production index; MCV, mean cell volume; MCH, mean cell hemoglobin; MCHC, mean cell hemoglobin concentration; sTfR, soluble transferrin receptor; IL-6, interleukin-6; hs-CRP, high sensitivity C-reactive protein; TNF, tumor necrosis factor; GDF15, growth differentiation factor 15.
