# Supplementary material for: Hepcidin and diabetes are independently related with soluble transferrin receptor levels in chronic dialysis patients
Source: Ren Fail. 2019 Jul 11;41(1):662–72. doi: 10.1080/0886022X.2019.1635893 (PMC6691825; doi:10.1080/0886022X.2019.1635893)
Supplement: Supplementary Table 1 [file IRNF_A_1635893_SM8842.docx]

**Supplementary Table 1** Hematological and biochemical data in dialysis patients according to the time of sample collection

|  | | Morning (*n* = 82) | | Midday (*n* = 88) | | Evening (*n* = 76) | |
| --- | --- | --- | --- | --- | --- | --- | --- |
| Hematological Data | |  | |  | |  | |
| Erythrocytes (x 10^12^/L) | | 3.75 (3.50 – 4.00) | | 3.80 (3.51 – 4.05) | | 3.65 (3.42 – 3.94) | |
| Hemoglobin (g/dL) | | 11.6 (11.0 – 12.1) | | 11.5 (11.0 – 12.2) | | 11.2 (10.5 – 12.2) | |
| Hematocrit (%) | | 35.6 (33.4 – 37.3) | | 35.4 (33.9 – 37.4) | | 34.4 (32.0 – 36.8) | |
| Reticulocytes (x 10^9^/L) | | 38.8 (26.6 – 56.0) | | 42.5 (26.6 – 58.9) | | 38.7 (26.5 – 59.5) | |
| RPI | | 0.70 (0.48 – 1.06) | | 0.73 (0.48 – 1.16) | | 0.63 (0.46 – 1.04) | |
| MCV (fL) | | 94.9 ± 5.4 | | 94.1 ± 5.0 | | 94.4 ± 5.9 | |
| MCH (pg) | | 30.8 (29.6 – 31.8) | | 30.6 (29.6 – 31.9) | | 31.2 (30.2 – 32.0) | |
| MCHC (g/dL) | | 32.6 (31.7 – 33.1) | | 32.4 (31.8 – 33.2) | | 32.6 (32.0 – 33.3) | |
| Platelets (x 10^9^/L) | | 187 (153 – 228) | | 189 (158 – 221) | | 208 (178 – 248) | |
| Leukocytes (x 10^9^/L) | | 6.1 (5.1 – 7.2) | | 6.0 (5.2 – 7.4) | | 6.5 (5.5 – 8.2) | |
| Iron metabolism markers | |  | |  | |  | |
| Iron (µg/dL) | | 60.0 (48.8 – 77.5) | | 53.0 (44.2 – 73.0) | | 53.0 (41.8 – 73.5) | |
| Transferrin (mg/dL) | | 184.0 (168.0 – 212.2) | | 182.0 (161.2 – 221.2) | | 193.5 (167.0 – 223.0) | |
| Transferrin saturation (%) | | 23.7 (19.2 – 29.3)* | | 20.8 (13.6 – 26.9) | | 19.8 (14.9 – 27.1) | |
| sTfR (nmol/L) | | 22.6 (16.9 – 28.4) | | 23.0 (17.0 – 28.8) | | 20.4 (16.1 – 27.2) | |
| Ferritin (ng/mL) | | 356.0 (212.0 – 491.5) | | 263.0 (167.2 – 408.2) | | 298.5 (183.5 – 463.0) | |
| Hepcidin (ng/mL) | | 64.2 (40.4 – 118.2) | | 79.6 (40.2 – 136.2) | | 79.6 (39.4 – 145.4) | |
| Inflammatory markers | |  | |  | |  | |
| IL-6 (pg/mL) | 4.31 (2.84 – 7.48) | | 3.84 (2.48 – 7.53) | | 3.95 (2.56 – 7.32) | |  |
| hs-CRP (mg/dL) | 0.26 (0.16 – 0.51) | | 0.41 (0.14 – 0.78) | | 0.52 (0.19 – 0.98) | |  |
| TNF-α (pg/mL) | 3.04 (2.65 – 4.11) | | 3.42 (2.70 – 4.36) | | 3.52 (2.53 – 5.27) | |  |
| GDF15 (pg/mL) | 11,400 (8,020 – 13,520) | | 11,360 (8,910 – 14,430) | | 12,160 (9,200 – 15,300) | |  |

Values are presented as mean ± SD or median (interquartile range). RPI, reticulocyte production index; MCV, mean cell volume; MCH, mean cell hemoglobin; MCHC, mean cell hemoglobin concentration; sTfR, soluble transferrin receptor; IL-6, interleukin-6; hs-CRP, high sensitivity C-reactive protein; TNF, tumor necrosis factor; GDF15, growth differentiation factor 15. *Significantly different from midday group (*P* = 0.033).
